# QTLs Associated with Agronomic Traits in the Cutler × AC Barrie Spring Wheat Mapping Population Using Single Nucleotide Polymorphic Markers

**DOI:** 10.1371/journal.pone.0160623

**Published:** 2016-08-11

**Authors:** Enid Perez-Lara, Kassa Semagn, Hua Chen, Muhammad Iqbal, Amidou N’Diaye, Atif Kamran, Alireza Navabi, Curtis Pozniak, Dean Spaner

**Affiliations:** 1 Department of Agricultural, Food and Nutritional Science, 4–10 Agriculture-Forestry Centre, University of Alberta, Edmonton, AB T6G 2P5, Canada; 2 Crop Development Centre and Department of Plant Sciences, University of Saskatchewan, 51 Campus Drive, Saskatoon, SK S7N 5A8, Canada; 3 Seed Centre, Department of Botany, The University of Punjab, New Campus, Lahore, 54590, Pakistan; 4 Department of Plant Agriculture, Crop Science Building, University of Guelph, Guelph, ON N1G 2W1, Canada; 5 National Institute for Genomics and Advanced Biotechnology, National Agricultural Research Centre, Park Road, Islamabad 45500, Pakistan; USDA-ARS Southern Regional Research Center, UNITED STATES

## Abstract

We recently reported three earliness *per se* quantitative trait loci (QTL) associated with flowering and maturity in a recombinant inbred lines (RILs) population derived from a cross between the spring wheat (*Triticum aestivum* L.) cultivars ‘Cutler’ and ‘AC Barrie’ using 488 microsatellite and diversity arrays technology (DArT) markers. Here, we present QTLs associated with flowering time, maturity, plant height, and grain yield using high density single nucleotide polymorphic (SNP) markers in the same population. A mapping population of 158 RILs and the two parents were evaluated at five environments for flowering, maturity, plant height and grain yield under field conditions, at two greenhouse environments for flowering, and genotyped with a subset of 1809 SNPs out of the 90K SNP array and 2 functional markers (*Ppd-D1* and *Rht-D1*). Using composite interval mapping on the combined phenotype data across all environments, we identified a total of 19 QTLs associated with flowering time in greenhouse (5), and field (6) conditions, maturity (5), grain yield (2) and plant height (1). We mapped these QTLs on 8 chromosomes and they individually explained between 6.3 and 37.8% of the phenotypic variation. Four of the 19 QTLs were associated with multiple traits, including a QTL on 2D associated with flowering, maturity and grain yield; two QTLs on 4A and 7A associated with flowering and maturity, and another QTL on 4D associated with maturity and plant height. However, only the QTLs on both 2D and 4D had major effects, and they mapped adjacent to well-known photoperiod response *Ppd-D1* and height reducing *Rht-D1* genes, respectively. The QTL on 2D reduced flowering and maturity time up to 5 days with a yield penalty of 436 kg ha^-1^, while the QTL on 4D reduced plant height by 13 cm, but increased maturity by 2 days. The high density SNPs allowed us to map eight moderate effect, two major effect, and nine minor effect QTLs that were not identified in our previous study using microsatellite and DArT markers. Results from this study provide additional information to wheat researchers developing early maturing and short stature spring wheat cultivars.

## Background

Global hexaploid wheat (*Triticum aestivum* L.) production increased from 627 million t in 2005 to 729 million t in 2014 (http://faostat3.fao.org). Canada is the seventh largest wheat-producing and the second largest wheat-exporting country. Average wheat yield in Canada has increased from 2.7 t ha^-1^ in 2005 to 3.1 t ha^-1^ in 2014, which is equivalent to an average yield increment of 35.7 kg ha^-1^ yr^-1^. Diseases and drought contribute to substantial reductions in overall wheat yields in Canada. Wheat breeders there aim to develop short, early maturing cultivars, with high grain yield and protein content, combined with resistance to major diseases, such as leaf, stem and yellow rusts caused by *Puccinia* sp., fusarium head blight caused by *Fusarium graminearum* and common bunt caused by both *Tilletia tritici* and *T*. *laevis* (http://www.pgdc.ca).

Wheat breeders, in addition to phenotypic selection, employ molecular markers in their breeding programs for different purposes, including parental selection, quality control analysis of advanced lines (cultivars) on genetic purity and identity, and for marker-assisted selection (MAS) [1]. The use of molecular markers in MAS requires identification of a subset of markers that are significantly associated with one or more genes or quantitative trait loci (QTLs) that regulate the expression of a trait of interest [2]. Both linkage-based QTL analysis and association mapping can be used to identify significant marker-trait associations, with each method having its own strength and weaknesses. Linkage-based QTL analysis depends on well-defined populations derived by crossing two parents with contrasting phenotype, which includes F_2_ or their derivatives, backcross, doubled haploid (DH), recombinant inbred lines (RILs), and near isogenic lines (NILs) [2, 3]. In wheat, RIL, NIL and DH are the most commonly used mapping populations, because they are homozygous and can be exchanged between different collaborators for both phenotyping and genotyping purposes [3]. However, NILs and RILs require long time and/or high cost to develop, and (ii) both populations only detect the additive effect but do not provide any information on dominant effect for any QTL [2, 4].

Several mapping studies associated with grain yield and other agronomic traits have been conducted for many years [5–12]. Recently, our group mapped QTLs associated with flowering, maturity, plant height and grain yield in a RIL population derived from the cross of two spring wheat cultivars, ‘Cutler’ and ‘AC Barrie’ [8]. The population was phenotyped in replicated field trials in four environments between 2007 and 2011, and genotyped with 488 microsatellite or simple sequence repeat (SSR) and diversity arrays technology (DArT) markers. That study uncovered seven QTLs on chromosomes 1B, 1D, 4A and 5B, of which only three QTLs were associated with the phenotypic data combined across all four environments. This included *QEps*.*dms-1B1* for both flowering and maturity, *QEps*.*dms-1B2* for maturity and *QEps*.*dms-5B1* for flowering time. One drawback of that study was low marker density (low genome coverage), which varied from 2 on chromosomes 4D and 6D to 57 on chromosome 2B, with an average of 23 markers per chromosome. DArT markers enable the simultaneous typing of several hundred polymorphic loci spread over the genome [13–15], but the dominant inheritance (present *vs*. absent variation) of DArT markers is one of the major drawbacks, as homozygous dominant and heterozygous individuals cannot be easily identified. SSR markers are widely used by wheat researchers for different reasons, including wide availability, co-dominant inheritance, multiallelism, high polymorphism, uniform distribution, and high polymorphic information content [16, 17]. SSRs are considered repeatable across laboratories [17], but their repeatability depends on several factors, including the nature of the study (mapping or marker-assisted selection in biparental populations that basically have a maximum of two segregating alleles per loci vs. genetic diversity and association mapping studies in diverse set of germplasm that have multiple alleles per loci), marker repeat length (di-, tri-, tetra- or penta-nucleotides), and the fragment separation methods (agarose gels, polyacrylamide gels, gel-based DNA sequencers or capillary DNA sequencers). Furthermore, most *Taq* DNA polymerases used for DNA synthesis add an extra base (usually an adenine) at the end of the amplified fragments [18], which generate spurious bands or peaks [19]. Hence, SSR markers produced by different laboratories or the same lab at different times using DNA sequencers are often difficulty in comparing, due to inconsistencies in allele size calling caused by the large variety of automatic DNA sequencing machines used for fragment analyses, each providing different migration, fluorescent dyes, and allele calling software [20]. Furthermore, SSR markers have lower throughput as compared with the highly multiplexed single nucleotide polymorphism (SNP) genotyping platforms and genotyping by sequencing [21]. Currently, SNP have become very popular for a wide range of applications due to high potential for automation that allows low cost and high throughput genotyping, high genomic coverage, co-dominant inheritance, and low genotyping error rates [22–24]. A total of 81,587 (90K) gene-associated SNPs are available for wheat researchers through the iSelect platform [25]. The consensus genetic position of 43,999 of the 90K SNPs was determined using eight mapping populations [25], which has provided a tremendous opportunity for wheat researchers conducting research requiring high marker density. The objectives of the present study were therefore to 1) identify genomic regions associated with flowering time under greenhouse and field conditions, and maturity, plant height and grain yield under field conditions in the ‘Cutler’ × ‘AC Barrie’ RIL population using the 90K Illumina iSelect SNP array; and 2) compare the results with a previous study on the same population using 488 microsatellite and DArT markers.

## Materials and Methods

### Plant material and phenotyping

The present study was based on a subset of 158 of the 177 RILs used in our previous study [8]. The RILs were derived from F_6:7_ at the University of Alberta using a single seed descent approach from a cross between two spring wheat cultivars, ‘Cutler’ and ‘AC Barrie’ [26]. ‘AC Barrie’ is characterized as having high protein content, late maturity (compared to ‘Cutler’) and resistance to some diseases [27]. ‘Cutler’ is an early maturing and semi-dwarf cultivar from the Canadian Prairie Spring class, and possesses the dominant *Vrn-A1a*, recessive *vrn-B1* and *vrn-D1* vernalization alleles at *Vrn1* loci, and the photoperiod insensitive allele *Ppd-D1a*. ‘AC Barrie’ possesses the same vernalization genes as the ‘Cutler’ with the photoperiod sensitive allele *Ppd-D1b*. ‘Cutler’ and ‘AC Barrie’ have the mutant *Rht-D1b and* wild type *Rht-D1a* alleles, respectively.

The 158 RILs and the two parents were evaluated five times for flowering time, maturity, plant height and grain yield under field conditions and twice for flowering time under greenhouse conditions [8]. Briefly, the RIL population and the two parents were phenotyped under field conditions between May and September in 2007, 2008 (planted on 7^th^ May and 4^th^ June), 2011 and 2012 at the University of Alberta South Campus Crop Research facility (53°19’N, 113°35’W), Edmonton, Canada. Seeds from the F_6:7_ were initially used for phenotyping in 2007; subsequent phenotyping trials were conducted using seeds multiplied from bulk harvest of typical heads of the previous year. Each field trial was conducted in a randomized incomplete block design with two to three replications depending on seed availability. Each entry was planted in 1.35 x 2.0 m double rows in 2007 and 2008, and 1.35 x 1.8 m with six rows in 2011 and 2012; row spacing in all field trials was 22.5 cm. All field trials were conducted in rainfed conditions using standard agronomic and cultural practices recommended for the station. Each RIL was evaluated for number of days to flowering and maturity, plant height and grain yield. As described in our previous study [8], number of days to flowering was recorded when 50% of the plants in any plot flowered (i.e., when 50% of spikes in an plot had completely emerged out of the flag leaf), while maturity was determined when 50% of the peduncles in a plot had completely lost green color. Days to flowering and maturity were converted into growing degree days by summing the average daily temperatures (over a base temperature of 0°C) from the date of seeding to the date when flowering or maturity was recorded [8]. The RILs and the parents were also evaluated for flowering time in a randomized incomplete block design with four replications under greenhouse conditions in 2006 and 2008 as described in our previous study [8]. All except the 2012 phenotype data used in the present study are the same as our previous study.

### DNA extraction and genotyping

Genomic DNA was extracted from three weeks old seedlings using a modified Cetyl Trimethyl Ammonium Bromide (CTAB) method [28]. DNA concentration was measured using a NanoDrop ND-1000 Spectrophotometer (Thermo Scientific, USA), and normalized to about 100 ng/μL. DNA sample were genotyped at the University of Saskatchewan Wheat Genomics lab, Saskatoon, Canada, with the 90K Illumina iSelect SNP array [25]. Alleles were called with the Illumina Genome Studio Polyploid Clustering version 1.0 software (Illumina, San Diego, USA) using default clustering parameters. Initially, all SNPs with more than three clusters were excluded from scoring. Because of the polyploidy nature of bread wheat, it was impossible to determine the actual nucleotide variant(s) in the 90K that are responsible for the observed polymorphism, because the actual variant calls may be influenced by off target variants (i.e., a cluster does not necessarily mean variant based on the source sequence file). Thus, additional filtering was done to select only those SNPs that segregated in a biallelic pattern and that were known to be allelic based on co-segregation with data from multiple mapping populations available to our programs.

We also genotyped the RIL population and the parents with *Ppd-D1 [29]* and *Rht-D1 [30]* at the Agricultural Genomics and Proteomics lab, University of Alberta, Edmonton, Canada. PCR was performed in 96-well plates in a total reaction volume of 10 μL that consisted of 50 ng template DNA, 1× magnesium-free PCR buffer, 1.5 mM MgCl_2_, 0.50 μM of each of the forward and reverse primer, 0.20 mM of each dNTP, and 1 unit GoTaq® Flexi DNA polymerase. All PCR components were purchased from Promega, USA. PCR amplifications were performed using Gene-Amp PCR System 9600 (PE-Applied Biosystems) as follows: 3 min initial denaturation at 94°C, followed by 40 cycles of 94°C for 30 sec, 60°C for 30 sec and 72°C for 30 sec, and a final extension of 7 min at 72°C. PCR fragments were separated with QIAxcel Advanced (Qiagne, USA) as described in the user’s manual using fast analysis kit (with 50 bp to 1.5 kb QX DNA size marker, 15 bp to 3 kb QX alignment marker, and DM150 analysis method).

### Statistical analyses

Least square means, F statistics and heritability were obtained using PROC MIXED and PROC IML in SAS version 9.3 (SAS Institute Inc. Cary, USA). We analysed each trial (environment) separately and then combined across all environments. Genotypes were considered fixed, while replications, blocks and years were considered random. For each trait, both test for normality and the frequency distribution were done using MiniTab v14. All SNPs that were monomorphic between the two parents and those with >20% missing data were excluded from linkage mapping. Linkage maps for the remaining SNPs and the two functional markers (*Ppd-D1* and *Rht-D1*) were constructed in two steps. First, ‘draft’ linkage maps were generated using the minimum spanning tree map (MSTMap) software [31] using a stringent cut off p-value of 1^−10^ and a maximum distance between markers of 15 cM. Second, the ‘draft’ maps were refined using the MapDisto version 1.7.5 software [32] using a cut off recombination value of 0.35, a minimum LOD score of 3.0 and Kosambi mapping function [33]. The best order of markers was generated using both “AutoCheckInversions” and “AutoRipple” commands. Linkage groups were assigned to chromosomes based on existing high density SNP maps of wheat [25, 34, 35]. For each SNP, chromosome arm was inferred from the draft sequence of the hexaploid wheat [36].

Composite interval mapping (CIM) was performed on the least square means of each trait using PLABQTL version 1.2 [37] with the following parameters: a minimum LOD score of 3.0, automatic cofactor selection, walking speed of 1 cM, a model to determine additive effects at individual QTL and additive x additive epistatic interactions, and F-to-Enter value of ten [38]. Additive effect is half the difference between the genotypic values of the two parents and the sign of the additive effect was used to identify the parental origin of the favorable alleles. In addition, we also computed the difference in phenotypic values of all RILs that had the ‘Cutler’ alleles at the two flanking markers of each QTL and those RILs that had the ‘AC Barrie’ alleles. QTL names were designated following the International Rules of Genetic Nomenclature (http://wheat.pw.usda.gov/ggpages/wgc/98/Intro.htm), which consisted of trait acronym, lab designation (dms = Dean Michael Spaner), and chromosome. In this study, QTLs that explained <10%, 10–20% and >20% of the total phenotypic variation (R^2^) were arbitrarily classified into minor, moderate and major effect QTLs, respectively. Genetic maps and QTL graphs were drawn using MapChart v2.1 [39].

## Results

### Summary of the phenotypic traits and markers

**Fig 1:**
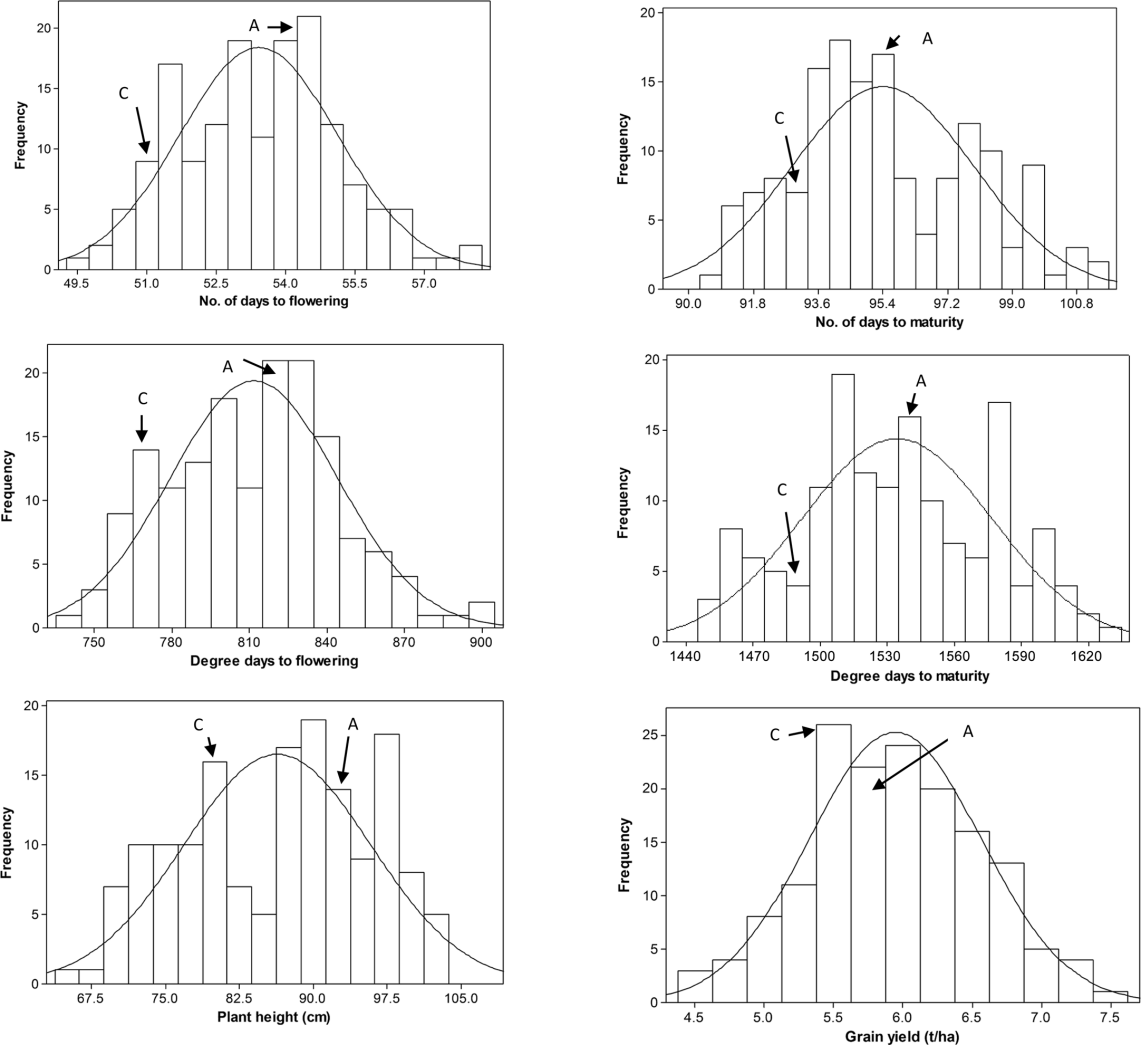
Frequency distribution of least square means computed from the combined data of five environments. The arrows indicate values of the two parents (C = Cutler; A = AC Barrie). ‘Cutler’ flowered and matured 2.6 days earlier, 12.9 cm shorter but produced 154.9 kg ha^-1^ lower grain yield than ‘AC Barrie’.

‘Cutler’ flowered/matured 2.6 days earlier and was 12.9 cm shorter, but produced 154.9 kg ha^-1^ lower grain yield than ‘AC Barrie’. The average plant height among the 158 RILs varied from 66 to 104 cm, and required between 49 and 58 days for flowering and between 91 and 101 days for maturity. Mean grain yield of the RILs varied between 4.6 and 7.4 t ha^-1^ (S1 Table). Broad sense heritability was 0.27 for grain yield, 0.43 for number of days to flowering, 0.48 for degree days to flowering, 0.50 for number of days to maturity, 0.46 for degree days to maturity, and 0.80 for plant height (S1 Table). Analysis of variance showed significant (p < 0.001) differences among genotypes for all traits (S1 Table). The distribution of least square means estimated from the combined phenotype data of all environments was normal (P ≥ 0.073) for flowering time, maturity, and grain yield. However, the Shapiro-Wilk test rejected the hypothesis of normality (P = 0.010) for plant height, which showed approximately bimodal distribution (Fig 1) than a more quantitative frequency distribution.

**Fig 2:**
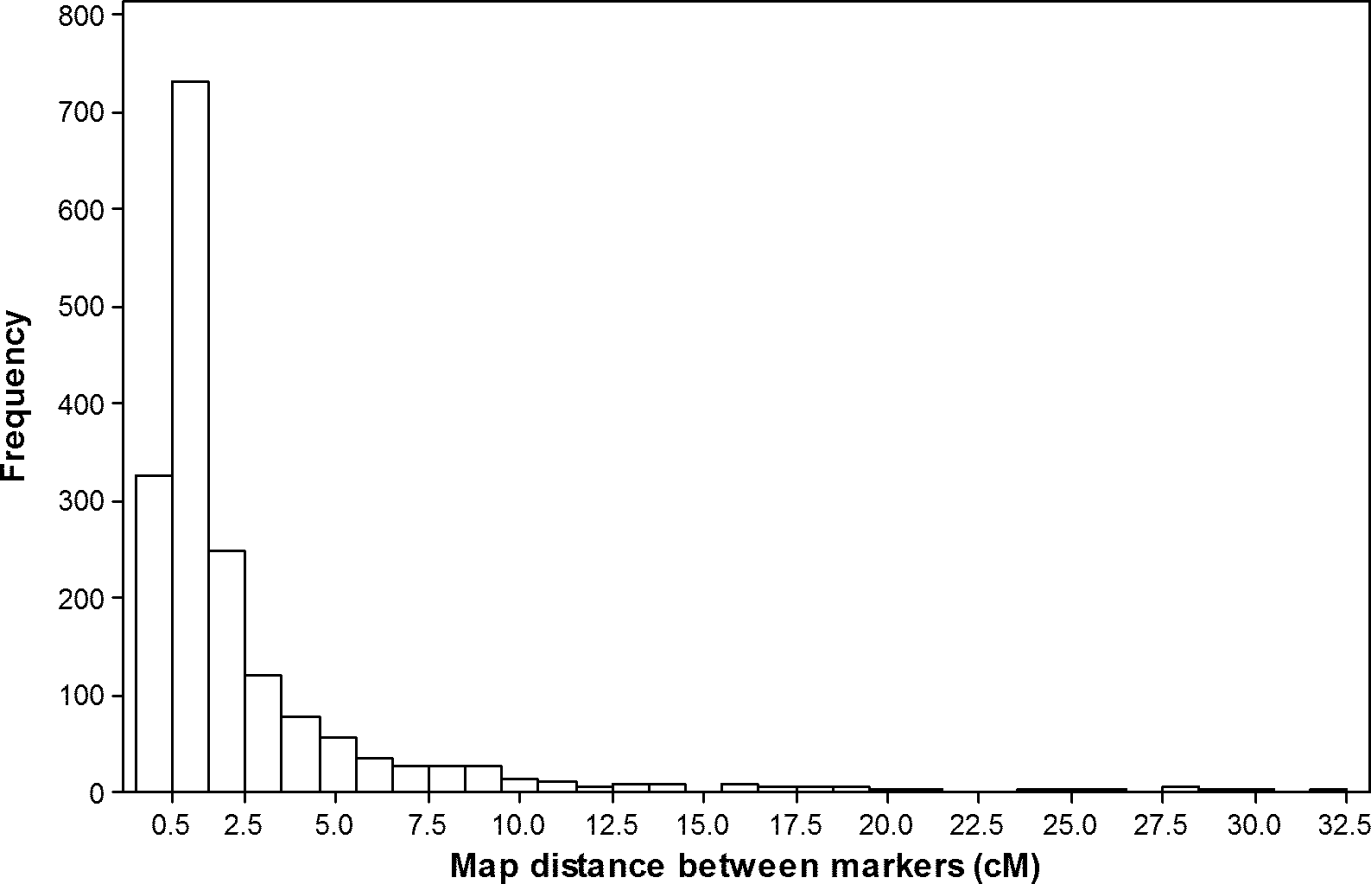
Observed frequency distribution of linkage map distances between adjacent loci based on the 1811 markers mapped to the 21 hexaploid wheat chromosomes.

Among the 81,587 SNPs used for genotyping the RIL population, approximately 87% of the SNPs (71,245 out of the 81,587 SNPs) were discarded for a number of reasons, including lack of polymorphism between the two parents, high (>20%) missing data, very high segregation distortion, and lack of linkage with other markers. The remaining 10,342 SNPs (12.7%) were incorporated in to the genetic linkage maps of the 21 chromosomes (Table 1, S2 Table). However, many SNPs co-segregated (mapped at exactly the same position), so they were excluded from the final dataset. Hence, only 1,809 of the 81,587 SNPs (2.2%) and two gene-based functional markers (*Rht-D1b* and *Ppd-D1a*) were used for QTL analyses, which is summarized in Table 1. The number of markers retained for QTL mapping varied from 9 on 5D to 221 on 5B, with an average of 86 markers per chromosome. The total map length across the 21 chromosomes was 3996 cM, with each chromosome varying in length from 22.3 cM on 5D to 373.7 cM on 5A. Map distance between adjacent markers (inter-marker interval) varied from 0.1 to 32.5 cM (Fig 2) and the overall average was 2.2 cM.

**Table 1:** Summary of the polymorphic markers integrated in to the 21 chromosomes based on 158 recombinant inbred lines derived from a cross between two spring wheat cultivars, ‘Cutler’ x ‘AC Barrie’. Chromosome 2A, 3A, 3B, 5A and 7B each has two linkage groups.

Linkage group	Total number of mapped markers	Total map length (cM)	No. of markers used for mapping	Map length used for mapping (cM)	Mean map distance/Marker
1A	429	178.2	101	178.2	1.8
1B	693	212.7	124	212.7	1.7
1D	264	118.4	46	118.4	2.6
2A	414	287.4	111	287.4	2.6
2A_LG2	54	11.9	7	11.9	1.7
2B	1539	229.3	173	229.3	1.3
2D	245	116.0	15	97.443	6.5
3A	453	261.2	83	261.2	3.1
3A_LG2	61	65.4	23	65.4	2.8
3B	586	150.6	107	150.6	1.4
3B_LG2	124	140.4	22	140.4	6.4
3D	45	96.4	30	96.4	3.2
4A	538	237.2	121	237.2	2.0
4B	471	244.2	91	244.2	2.7
4D	47	125.1	18	74.848	4.2
5A	432	254.6	72	254.6	3.5
5A_LG2	106	119.1	36	119.1	3.3
5B	1438	214.2	221	214.2	1.0
5D	37	22.3	9	22.3	2.5
6A	523	200.2	73	200.2	2.7
6B	440	229.0	67	229	3.4
6D	76	43.0	9	43	4.8
7A	737	201.8	145	201.8	1.4
7B	313	124.5	52	124.5	2.4
7B_LG2	190	44.0	21	44	2.1
7D	89	137.7	34	137.7	4.1
Total	10,344	4,064.8	1,811	3,996.0	

### QTL analyses

**Fig 3:**
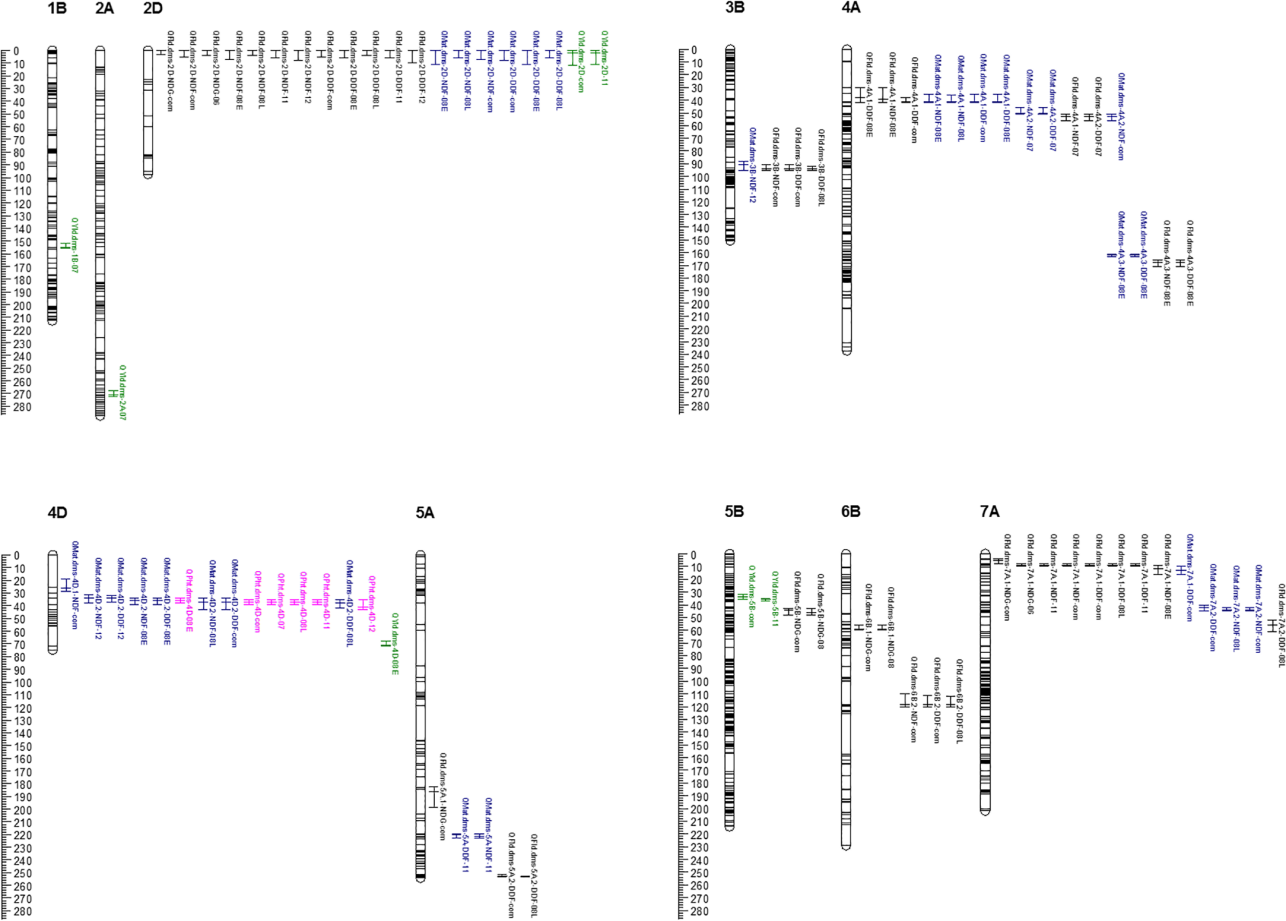
Linkage map of the 10 wheat chromosomes that have at least one QTL associated with flowering time, maturity, plant height and/or grain yield. Map position in centiMorgans (cM) is shown on the left side of the chromosomes, with each horizontal line representing a marker. QTLs names are shown on the right side of each linkage group, with bars indicating their confidence interval. QTLs for flowering, maturity, plant height and grain yield are in black, red, pink and green font, respectively. QTLs for flowering time under greenhouse have NDG-06, NDG-08 and NDG-com after chromosome number, which refers to the trials in 2006, 2008 and combined, respectively. QTLs for both flowering and maturity under field conditions have either NDF or DDF, which refers to number of days under field and degree days under field, respectively, followed by 07, 08E (early plating), 08L (late planting), 11, 12 or com after chromosome, which indicate the last two digits of the corresponding year of the trials or combined data of all trials. QTLs for pant height and grain yield have the last two digits of the trial year or com for combined. See S3 Table for details.

Composite interval mapping (CIM) was performed on the least square means estimated for individual environments and also combined across two environments for flowering time under greenhouse and five environments for flowering time, maturity, plant height and grain yield under field conditions. Table 2 shows summary of the QTLs for the combined environments, while both Fig 3 and S3 Table show detailed results for both single and combined environments. We found 5 QTLs associated with the two years combined flowering time data under greenhouse, which altogether explained 73.1% of the phenotypic variance. The five QTLs for flowering time under greenhouse mapped at the proximal tip of chromosome 2DS (*QFlt*.*dms-2D*), at 187 cM on 5A (*QFlt*.*dms-5A*.*1*), at 44 cM on 5B (*QFlt*.*dms-5B*), at 59 cM on 6B (*QFlt*.*dms-6B*.*1*) and at 5 cM on 7A (*QFlt*.*dms-7A*.*1*). The proportion of phenotypic variance explained by each flowering time QTL under greenhouse varied from 6.9% for *QFlt*.*dms-5A*.*1* to 36.6% for *QFlt*.*dms-2D*. *QFlt*.*dms-2D* is the only major effect QTL for flowering time under greenhouse, flanked by the known photoperiod insensitive allele *Ppd-D1a* and SNP marker (wsnp_CAP11_c3842_1829821) (Table 2). For the combined phenotype data across all five environments conducted under field conditions, there were 4–6 QTLs for flowering time, 4–5 QTLs for maturity, one QTL for plant height, and two QTLs for grain yield (Table 2, Fig 3). We found four QTLs (*QFlt*.*dms-2D*, *QFlt*.*dms-3B*, *QFlt*.*dms-6B*.*2* and *QFlt*.*dms-7A*.*1*) associated with the number of days to flowering and six QTLs (*QFlt*.*dms-2D*, *QFlt*.*dms-3B*, *QFlt*.*dms-6B*.*2*, *QFlt*.*dms-7A*.*1*, *QFlt*.*dms-4A*.*1* and *QFlt*.*dms-5A*.*2*) for degree days to flowering. Four of the QTLs for flowering time under field conditions (*QFlt*.*dms-2D*, *QFlt*.*dms-3B*, *QFlt*.*dms-6B*.*2* and *QFlt*.*dms-7A*) were common between the number of days and degree days. For maturity, we found four QTLs (*QMat*.*dms-2D*, *QMat*.*dms-4A*.*2*, *QMat*.*dms-4D*.*1* and *QMat*.*dms-7A*.*2*) for the number of days to maturity and five QTLs (*QMat*.*dms-2D*, *QMat*.*dms-4A*.*1*, *QMat*.*dms-4D*.*2*, *QMat*.*dms-7A*.*1* and *QMat*.*dms-7A*.*2*) for maturity in degree days, but only two QTLs (*QMat*.*dms-2D* and *QMat*.*dms-7A*.*2*) were common between the two datasets. To account for the difference in temperature across years, therefore, we only present details for the QTLs associated with flowering time and maturity in growing degree days.

**Table 2:** Summary of the QTLs associated with flowering time, maturity, plant height and grain yield on 158 recombinant inbred lines derived from a cross between spring wheat cultivars, ‘Cutler’ and ‘AC Barrie’. The results are for the combined two environments for flowering time under greenhouse and five environments for flowering time, maturity, plant height and grain yield under field conditions.

QTL	Trait*	Condition	Chrom	Position (cM)	Confidence interval (cM)	Left marker	Right marker	LOD	Partial R^2^ (%)	Additive effect**	Difference***
*QFlt*.*dms-2D*	Flowering (days)	GH	2D	0	0–3	Ppd-D1a	wsnp_CAP11_c3842_1829821	16.3	36.6	-2.6	-5.4
*QFlt*.*dms-5A*.*1*	Flowering (days)	GH	5A	187	183–199	Kukri_c20258_143	JD_c3525_1503	3.3	6.9	0.9	2.0
*QFlt*.*dms-5B*	Flowering (days)	GH	5B	44	43–48	BS00063785_51	IACX5818	5.0	12.3	-1.1	-1.8
*QFlt*.*dms-6B*.*1*	Flowering (days)	GH	6B	59	56–60	Tdurum_contig11700_1247	wsnp_Ra_c2730_5190365	4.2	10.0	-1.1	-2.0
*QFlt*.*dms-7A*	Flowering (days)	GH	7A	5	4–8	Excalibur_c16355_712	RAC875_c18446_521	3.7	7.3	-1.0	-1.4
*QFlt*.*dms-2D*	Flowering (days)	Field	2D	0	0–5	Ppd-D1a	wsnp_CAP11_c3842_1829821	7.2	19.6	-0.8	-1.1
*QFlt*.*dms-3B*	Flowering (days)	Field	3B	94	91–95	Excalibur_c45968_83	CAP12_rep_c7901_114	5.8	13.5	0.6	1.0
*QFlt*.*dms-6B*.*2*	Flowering (days)	Field	6B	118	110–120	wsnp_Ex_c4124_7455225	Kukri_c49331_77	3.1	6.7	-0.4	-0.7
*QFlt*.*dms-7A*	Flowering (days)	Field	7A	9	8–10	Tdurum_contig11613_329	wsnp_Ex_c30239_39179460	4.7	12.7	-0.5	-0.9
*QFlt*.*dms-2D*	Flowering (DD)	Field	2D	0	0–5	Ppd-D1a	wsnp_CAP11_c3842_1829821	7.8	25.4	-14.2	-21.0
*QFlt*.*dms-3B*	Flowering (DD)	Field	3B	94	91–95	Excalibur_c45968_83	CAP12_rep_c7901_114	6.7	10.7	10.6	19.7
*QFlt*.*dms-4A*	Flowering (DD)	Field	4A	41	38–42	CAP12_rep_c4000_432	wsnp_Ex_c54453_57331510	3.7	6.3	-7.6	-17.6
*QFlt*.*dms-5A*.*2*	Flowering (DD)	Field	5A	253	252–254	Tdurum_contig86202_175	wsnp_Ra_c10915_17838202	3.7	7.5	7.5	16.1
*QFlt*.*dms-6B*.*2*	Flowering (DD)	Field	6B	118	111–120	wsnp_Ex_c4124_7455225	Kukri_c49331_77	3.4	9.3	-7.4	-13.8
*QFlt*.*dms-7A*	Flowering (DD)	Field	7A	9	8–10	Tdurum_contig11613_329	wsnp_Ex_c30239_39179460	6.4	15.6	-10.3	-15.9
*QMat*.*dms-2D*	Maturity (days)	Field	2D	0	0–7	Ppd-D1a	wsnp_CAP11_c3842_1829821	3.1	11.2	-0.7	-1.3
*QMat*.*dms-4A*.*2*	Maturity (days)	Field	4A	53	51–56	Ra_c7973_1185	wsnp_Ex_c10390_17007929	3.5	6.5	-0.7	-1.2
*QMat*.*dms-4D*.*1*	Maturity (days)	Field	4D	26	19–29	Excalibur_c5010_1336	Kukri_rep_c68594_530	4.9	13.4	0.8	1.6
*QMat*.*dms-7A*.*2*	Maturity (days)	Field	7A	44	42–45	RAC875_c14982_577	Tdurum_contig20214_279	3.1	8.8	0.6	1.2
*QMat*.*dms-2D*	Maturity (DD)	Field	2D	0	0–8	Ppd-D1a	wsnp_CAP11_c3842_1829821	3.9	10.4	-14.2	-21.2
*QMat*.*dms-4A*.*1*	Maturity (DD)	Field	4A	41	35–42	CAP12_rep_c4000_432	wsnp_Ex_c54453_57331510	3.1	12.0	-10.7	-22.7
*QMat*.*dms-4D*.*2*	Maturity (DD)	Field	4D	37	34–43	Rht-D1b	wsnp_CAP11_c356_280910	5.7	16.0	15.7	33.0
*QMat*.*dms-7A*.*1*	Maturity (DD)	Field	7A	13	10–16	wsnp_Ra_c63822_63288359	wsnp_BG313770A_Ta_2_3	3.0	9.1	-11.3	-11.2
*QMat*.*dms-7A*.*2*	Maturity (DD)	Field	7A	42	40–45	Tdurum_contig37154_190	RAC875_c14982_577	5.7	16.2	15.7	20.8
*QPht*.*dms-4D*	Plant height	Field	4D	37	35–39	Rht-D1b	wsnp_CAP11_c356_280910	16.2	37.8	-6.3	-13.2
*QYld*.*dms-2D*	Grain yield	Field	2D	2	0–12	Ppd-D1a	wsnp_CAP11_c3842_1829821	4.3	8.6	-248.2	-436.0
*QYld*.*dms-5B*	Grain yield	Field	5B	34	32–36	Excalibur_c30667_102	Ku_c6193_821	3.7	7.7	-184.0	-321.8

* Flowering (days) = number of days to 50% flowering under greenhouse (GH) or field conditions; Maturity (days) = number of days to maturity under field condition; Flowering (DD) = flowering time in degree days; Maturity (DD) = maturity in degree days.

** Additive effect is half the difference between the genotypic values of the two parents. For grain yield, negative additive effects indicate that the favorable alleles originated from ‘AC Barrie’. For flowering, maturity and plant height, negative additive effects indicate the opposite (the favorable alleles originated from ‘Cutler’).

***Difference = the difference in phenotypic values of all RILs that had the ‘Cutler’ alleles at both flanking markers of every QTL to those that had the ‘AC Barrie’ alleles. The units for differences are number of days or degree days for both flowering and maturity, cm for plant height, and kg ha^-1^ for grain yield.

The total phenotypic variance explained by all QTLs associated with flowering time and maturity in degree days, plant height and grain yield across the combined data of the five environments was 74.8, 63.7, 37.8 and 16.3%, respectively. All QTLs associated with each trait exhibited mainly additive effects and QTL by QTL interactions were negligible (R^2^ < 1.5%). The six QTLs associated with flowering time (in degree days) mapped at the proximal tip of 2DS (*QFlt*.*dms-2D*), at 94 cM on 3B (*QFlt*.*dms-3B*), at 41 cM on 4A (*QFlt*.*dms-4A*.*1*), at 253 cM on 5A (*QFlt*.*dms-5A*.*2*), at 118 cM on 6B (*QFlt*.*dms-6B*.*2*) and at 9 cM on 7A (*QFlt*.*dms-7A*.*1*). Each QTL individually explained between 6.3 and 25.4% of the phenotypic variance across the five environments (Table 2), with *QFlt*.*dms-2D* as the only major effect QTL for flowering time under field conditions. The favorable alleles for all flowering QTLs except *QFlt*.*dms-3B* and *QFlt*.*dms-5A*.*2* originated from ‘Cutler’. Lines that had the favorable alleles at the two flanking markers of each flowering QTL showed a reduction of 13.8 to 21.1 degree days on flowering time as compared with those lines that had the unfavorable alleles. When individual environments were considered (S3 Table), *QFlt*.*dms-2D* was consistently detected in four of the five environments, followed by *QFlt*.*dms-7A*.*1* in two environments (Fig 3); the other QTLs were identified only in a single environment.

The five QTLs associated with maturity in degree days across the combined data were located at the proximal tip of 2DS (*QMat*.*dms-2D*), at 41 cM on 4A (*QMat*.*dms-4A*.*1*), at 37 cM on 4D (*QMat*.*dms-4D*.*2*) plus at both 13 and 42 cM on 7A (*QMat*.*dms-7A*.*1* and *QMat*.*dms-7A*.*2*). Each maturity QTL explained between 9.1 and 16.2% of the phenotypic variance across all combined environments (Table 2). The favorable alleles for *QMat*.*dms-4D*.*2 and QMat*.*dms-7A*.*2* originated from ‘AC Barrie’, while those for *QMat*.*dms-2D*, *QMat*.*dms-4A*.*1* and *QMat*.*dms-7A*.*1* from ‘Cutler’. RILs that had the favorable alleles at the two flanking markers of each QTL showed a reduction in maturity from 11.2 to 33.0 degree days than those RILs that possessed the unfavorable alleles. When results from individual environments were considered, only *QMat*.*dms-2D* and *QMat*.*dms-4D*.*2* were detected in two and three environments, respectively (Fig 3, S3 Table); the other QTLs were detected either in a single environment or only in the combined environments (but not in any of the individual environments).

For plant height, we found a single major effect QTL that mapped at 37 cM on chromosome 4D (*QPht*.*dms-4D*), flanked by a height reducing *Rht-D1b* gene and *wsnp_CAP11_c356_280910*. This QTL had a LOD score of 16.2 and accounted for 37.8% of the phenotypic variance for plant height across the combined data of the five environments. RILs that had the ‘Cutler’ alleles at the two flanking markers were on average 13.2 cm shorter than those RILs that possessed the ‘AC Barrie’ alleles. When individual environments were considered, *QPht*.*dms-4D1* was consistently detected within the same confidence interval in all five individual environments (Fig 3, S3 Table). The LOD score and phenotypic variance explained by this QTL on individual environments varied from 13.2 to 18.8 and from 30.8 to 38.5%, respectively, which is equivalent to a reduction in plant height by 10.7 to 14.3 cm. For grain yield, we found two QTLs at the proximal tip on 2DS (*QYld*.*dms-2D*) and at 34 cM on 5B (*QYld*.*dms-5B*), which explained 7.7 to 8.6% of the phenotypic variance for grain yield across five environments. RILs that had the ‘AC Barrie’ alleles at the two flanking markers of *QYld*.*dms-2D* and *QYld*.*dms-5B* produced on average 436.0 and 321.8 kg ha^-1^ more grain yield than those RILs that had the ‘Cutler’ alleles. When individual environments were considered, each QTL was detected only in a single environment (Fig 3, S3 Table).

### Coincident QTLs

Four of the nineteen QTLs associated with the combined phenotype data of the four traits were common (coincident) for two or three traits. The first coincident QTL is the one that mapped at the proximal tip on chromosome 2DS, which is associated with flowering time both under greenhouse and field conditions (*QFlt*.*dms-2D*), maturity (*QMat*.*dms-2D*) and grain yield (*QYld*.*dms-2D*). As in our previous study [8], coincident QTLs for both flowering and maturity time belong to earliness *per se* QTL; hence, both *QFlt*.*dms-2D* vs *QMat*.*dms-2D are named QEps*.*dms-2D*. *QEps*.*dms-2D* explained 36.6% and 25.4% for flowering time under greenhouse and field conditions, respectively, 10.4% for maturity, and 8.6% for grain yield. RILs carrying the photoperiod insensitive mutant *Ppd-D1a* alleles from ‘Cutler’ at the two flanking markers for *QEps*.*dms-2D* have differed from those containing the photoperiod sensitive *Ppd-D1b* alleles from ‘AC Barrie’ for flowering time (p < 0.012), maturity (p < 0.050) and grain yield (p < 0.001), but not for plant height (S4 Table). On average, therefore, RILs that had the ‘Cutler’ alleles at the two flanking markers of the *QEps*.*dms-2D* flowered/matured 1.1–5.4 days earlier, but suffered a yield penalty of 436 kg ha^-1^ than those RILs that possessed the ‘AC Barrie’ alleles (Table 2). The second coincident QTL mapped at 41 cM on 4A and it was associated with both flowering time (*QFlt*.*dms-4A*.*1*) and maturity (*QMat*.*dms-4A*.*1*), here referred as *QEps*.*dms-4A*.*1*. RILs carrying the ‘Cutler’ alleles at the two flanking markers for *QEps*.*dms-4A*.*1* differed (p < 0.005) from those containing the ‘AC Barrie’ alleles for flowering time under field conditions and maturity, but not for plant height and grain yield (S4 Table). RILs that had the ‘Cutler’ alleles at the two flanking markers for *QEps*.*dms-4A*.*1* flowered/matured 17.6–22.7 degree days earlier than those RILs that had the ‘AC Barrie’ alleles (Table 2). The third coincident QTL was on 4D and it was associated with both maturity (*QMat*.*dms-4D*.*2*) and plant height (*QPht*.*dms-4D*). RILs carrying the ‘Çutler’ alleles at the two flanking markers of the coincident QTL on 4D showed significant differences (p < 0.001) with those containing ‘AC Barrie’ alleles for maturity and plant height, but not for flowering time and grain yield (S4 Table). RILs that consisted of the ‘Cutler’ alleles at the two flanking markers of *QMat*.*dms-4D*.*2* and *QPht*.*dms-4D* were 13.2 cm shorter, but took 33 degree days longer to mature than those RILs that possessed the ‘AC Barrie’ alleles (Table 2). Finally, the QTL that mapped between 8 and 16 cM on 7A was the third earliness *per se* QTL (*QEps*.*dms-7A*), associated with both flowering time (*QFlt*.*dms-7A*.*1*) and maturity (*QMat*.*dms-7A*.*1*), with RILs consisting of the ‘Cutler’ alleles at the two flanking markers showing a reduction in flowering/maturity by 11.2–15.9 degree days than those RILs that had the ‘AC Barrie’ alleles (Table 2). However, RILs carrying the ‘Çutler’ alleles at the two flanking markers for *QEps*.*dms-7A* were different from those containing ‘AC Barrie’ alleles only for flowering time under field conditions (p < 0.001), but not for maturity, plant height and grain yield (S4 Table).

## Discussion

### Comparison with our previous study

Based on 488 SSR and DArT markers, we previously reported three QTLs associated with the combined phenotypic data across four environments [8], which includes one coincident QTL for both flowering time and maturity at 31–33 cM on 1B (*QEps*.*dms-1B1*), one QTL for maturity at 36 cM on 1B (*QEps*.*dms-1B2*) and one QTL for flowering time at 76 cM on 5B (*QEps*.*dms-5B1*). That study failed to identify any QTL for both plant height and grain yield across the combined phenotypic data of the four environments; only two environment specific QTLs were reported for grain yield. Our previous study was based on a total map length of 2,279 cM, with individual chromosome varying from 36 to 229 cM; the overall average map distance among adjacent markers (inter-marker interval) was 4.7 cM. We thought that the low marker density might have restricted our ability to identify more QTLs with larger phenotypic effect. The use of larger number of polymorphic markers provides a more accurate overview of informative recombinations and greater saturation of genetic linkage maps. The denser the genetic maps, the lower the chance of missing true QTLs [40]. Our present study was based on 1809 polymorphic SNPs and two known gene-based functional markers (*Ppd-D1a* and *Rht-D1b*), which resulted in a total map length of 3996 cM and an overall average inter-marker interval of 2.2 cM. As compared with our previous study, therefore, the genome coverage in the present study increased by 78%, while average inter-marker interval decreased over two fold. Based on such higher genome coverage and reduction in map distance among adjacent markers, we expected to narrow down the confidence interval of the QTLs that we reported in our previous study and also uncover additional QTLs that may have been missed in our previous study. In the present study, we uncovered a total of nineteen QTLs associated with the combined phenotypic data, which includes five for flowering time in the greenhouse, six for flowering time in the field, five for maturity, one for plant height, and two for grain yield (Table 2). However, we only identified one environment specific QTL for grain yield on 1B (*QYld*.*dms-1B*) and one QTL for flowering time under greenhouse on 5B, but we were not sure whether these two QTLs mapped at the same confidence interval as the three QTLs (*QEps*.*dms-1B1*, *QEps*.*dms-1B2* and *QEps*.*dms-5B1*) reported in our previous study.

In order to verify the position of the QTLs identified on 1B and 5B in the two studies, we conducted QTL analyses using a genetic map constructed by combining DArT, SSR and SNP markers on chromosomes 1B and 5B. Only 131 out of the 158 RILs had a complete DArT, SSR and SNP genotypic data. The analyses conducted on genotypic and phenotypic data of 131 RILs and combined map of the three types of markers (DArT, SSRs and SNPs) identified one of the QTLs for maturity on 1B between 74.5 and 80.5 cM interval, which accounted for 9.7–13.1% of the phenotypic variance for maturity in degree days across the combined data plus the 2007 and 2011 environments (S5 Table). Although the genetic position for *QEps*.*dms-1B2* was different between the two studies (which is expected with addition of large number of SNPs into DArT and SSRs), one of the flanking DArT markers (wPt-2694) remained the same. However, the position of the QTL associated with the combined grain yield data across 5 environments (*QYld*.*dms-1B*) was 52 cM distal to wPt-2694, which suggests that the QTL for maturity is different from that of the QTL for grain yield. For the QTL on 5B, the analysis using combined DArT, SSR and SNP markers identified *QEps*.*dms-5B1*, which has been reported in our previous study [8]. In the present study, *QEps*.*dms-5B1* was associated with flowering time in the 2007 and maturity in the 2008 early planting environments (S5 Table). This QTL was flanked by two DArT markers (wPt-1304 and wPt-666939), and explained between 7.6 and 11.8% of the phenotypic variance for flowering time and maturity in the individual environments. In both the previous and present studies, wPt-666939 is one of the flanking markers for *QEps*.*dms-5B1*. Therefore, the inclusion of the DArT markers on both 1B and 5B allowed us to identify the QTLs for earliness *per se* that we failed to detect using the SNP markers alone. In addition, the inclusion of DArT markers has also helped us to uncover three additional QTLs on 5B, which includes one coincident QTL for grain yield and plant height at 194–204 cM interval and one QTL for plant height (S5 Table).

However, the integration of the SSR and DArT markers with the SNPs had two limitations. First, it reduced the number of RILs with complete genotypic and phenotypic data from 158 to 131. Secondly, the SSR and DArT markers affected locus order for many SNPs, which was difficult to tell due to lack of physical positions or consensus linkage maps for the different types of markers. Third, a number of SNPs remained either unlinked or fall into several smaller linkage groups, which significantly reduced the number of markers integrated in the linkage maps. We suspected an error in the DArT genotypic data, either mislabeling and/or data coding errors during linkage map construction and QTL analyses. The second possible reason may be the use of large numbers of DArT markers in our previous study, which are primarily dominant in inheritance [13, 41]. We therefore present only QTL results obtained using the SNPs and the two functional markers (*Ppd-D1a* and *Rht-D1b)*.

### Comparison with other studies

The QTLs for flowering time in the combined environments both under greenhouse and field conditions mapped on chromosomes 2D, 3B, 4A, 5A, 5B, 6B and 7A, each explaining between 6.3 and 36.6% of the phenotypic variance (Table 2). For maturity, we found QTLs on 2D, 4A, 4D and 7A, each explaining between 9.1 and 16.2% of the phenotypic variance across five environments. In a study conducted on four European winter wheat DH populations [42], the authors reported QTLs for flowering time on almost all the wheat chromosomes. In another Canadian western red spring wheat RIL population derived from a cross between ‘CDC Teal’ and ‘CDC Go’, our group has also recently reported a QTL associated with heading, flowering and maturity on chromosome 4A that accounted 8.9–20.2% of the phenotypic variance across three environments [10]. Several previous studies have reported genes and/or QTLs for both flowering time and maturity on both homoeologous group 5 [43, 44] and group 2 [45–48] chromosomes. In bread wheat, vernalization response is controlled by three *Vrn* loci (*Vrn-1*, *Vrn-2* and *Vrn-3*) of which *Vrn-A1*, *Vrn-B1 and Vrn-D1* mapped on the long arm of chromosomes 5A, 5B and 5D, respectively [49, 50]. The three *Vrn-1* genes directly influence both flowering and maturity [51, 52]. However, we are not sure whether the QTLs for flowering time that we mapped on both 5A and 5B are in the same positions as the *Vrn-1* genes, because (i) direct comparison of the genetic map positions across different studies is not possible without having either common set of markers or physical positions; (ii) “Cutler’ and ‘AC Barrie’ were monomorphic for the *VRN1* loci, both having the dominant *Vrn-A1a* and recessive *vrn-B1* and *vrn-D1* alleles [26].

In the present study, we found a major effect and coincident QTL on 2D for flowering time both under greenhouse and field conditions (*QFlt*.*dms-2D*), maturity (*QMat*.*dms-2D*) and grain yield (*QYld*.*dms-2D*). This coincident QTL is flanked by the well-known photoperiod response *Ppd-D1a* locus, and accounted from 19.6 to 36.6% for flowering time, from 10.4 to 11.2% for maturity, and 8.6% for grain yield (Table 2). A recent genome-wide associations study for heading date in a panel of diverse European winter cultivars reported highly significant marker-trait associations at *Ppd-D1* gene [48]. In wheat, photoperiod response is primarily controlled by the *Ppd-1* loci that mapped on the short arms of chromosomes 2D, 2B, and 2A and influences both flowering time and maturity [29, 46, 50, 53, 54]. In general, the *Ppd-D1* has been considered the strongest allele for photoperiod insensitivity, followed by *Ppd-B1* and *Ppd-A1* [47, 50, 55], but there are conflicting reports that suggests that *Ppd-B1a* could be as strong as *Ppd-D1* [45]. The favorable alleles for the flowering time/maturity and grain yield QTL on 2D originated from ‘Cutler’ and ‘AC Barrie’, respectively. If selection were to be made for the ‘Cutler’ alleles at all three traits, RILs carrying the mutant *Ppd-D1a* alleles from ‘Cutler’ at the two flanking markers showed significant (p <0.05) reduction on flowering/maturity, but suffered highly significant (p <0.001) yield penalty (i.e., it reduced grain yield by 436 kg ha^-1^). Coincident QTLs have been reported in several other studies [56–60], which could be due to (i) tight linkages between genes or QTLs that regulate the expression of separate traits, but the statistical method failed to discriminate them; or (ii) pleiotropic effect, the same gene or QTL may have an effect on two or more traits simultaneously [61]. In the present study, the genetic distance between the two flanking markers (*Ppd-D1a* and wsnp_CAP11_c3842_1829821) for the coincident QTL on 2D (*QFlt*.*dms-2D*, *QMat*.*dms-2D* and *QYld*.*dms-2D*) is 22.6 cM, which is too large. It is, therefore, highly likely that the chromosomal segments associated with this coincident QTL on 2D carry two or more genes or QTLs, which could be determined by screening larger numbers of recombinants to break up the linkage [62].

Although the ‘Cutler’ and ‘AC Barrie’ RIL population was primarily developed to study flowering time, maturity and photoperiodism [26], results from our studies showed that ‘Cutler’ matured 2.6 days earlier and 12.9 cm shorter, but produced 154.9 kg ha^-1^ lower yield than ‘AC Barrie’, which clearly suggests that the same population could also be used for mapping genomic regions associated with plant height and grain yield. Our previous study, however, failed to uncover QTLs for the combined plant height and grain yield data across four environments [8]. The present study identified a coincident major QTL for plant height (*QPht*.*dms-4D*) and medium effect QTL for maturity (*QMat*.*dms-4D*.*2*) on chromosome 4D (Table 2, Fig 3). *QPht*.*dms-4D* was consistently detected at the same confidence interval in all five individual environments and also combined across all environments, while *QMat*.*dms-4D*.*2* has been detected in the 2008 (both early and late planting), 2012 and combined environments (S3 Table). RILs carrying the ‘Çutler’ alleles at the two flanking markers of this coincident QTL on 4D showed highly significant differences (p < 0.005) with those containing ‘AC Barrie’ alleles for both maturity and plant height, but not for flowering time and grain yield. Depending on the data used for analyses (individual or combined environments), this coincident QTL explained from 30.9 to 38.5% and from 13.8 to 19.3% of the phenotypic variance for plant height and maturity, respectively. RILs with the ‘Cutler’ alleles at the two flanking markers were on average 10.7 to 14.3 cm shorter, but required from 30.5 to 82.3 more degree days to mature than those RILs with the ‘AC Barrie’ alleles. Traits that showed more quantitative frequency distributions with a single peak are believed to be controlled by several QTLs, each with moderate to small individual effects, as compared to a bimodal distribution that is predominantly controlled by a single gene, clusters of tightly linked genes or few major effect QTLs [63, 64]. The least squares means of plant height across the five environments showed skewness and bimodal distribution (Fig 1). It is not therefore unexpected to uncover a large effect genomic region associated with plant height with bimodal frequency distribution. One of the flanking markers for this coincident QTL on 4D is *Rht-D1b*, a well-known semi-dwarfing gene [30, 65]. In the combined data of the five environments, the *Rht-D1b* mutant allele was present in 54 RILs and absent in 78 RILs. In hexaploid wheat, dwarfing has been achieved mainly through the introduction of *Rht-B1b* on 4B and *Rht-D1b* on 4D [30, 65], which have been introduced in many cultivars grown worldwide [66]. A recent genome-wide association studies was conducted for plant height using a set European winter and spring wheat varieties evaluated across eight environments [67]. That study reported highly significant association between plant height and *Rht-D1*, which revealed the presence of *Rht-D1b* and *Rht-B1b* mutant alleles in 58% and 7% of the varieties, respectively.

The QTL on 4D that reduced plant height also increased days to maturity. As discussed above for the coincident QTL on 2D, coincident QTLs on 4D could also be due to either tight linkages between genes or QTLs or pleiotropic effect [61]. For example, one study fine mapped phenotypic effects segregating within a 1 cM chromosome interval in *Arabidopsis thaliana* for which lines with recombination breakpoints were available [68]. The authors found that the 1 cM chromosome interval contained two growth rate QTLs within 210 kb, which showed epistasis. In the present study, the two flanking markers (*Rht-D1b* and wsnp_CAP11_c356_280910) for the coincident QTL on 4D (*QMat*.*dms-4D*.*2* and *QPht*.*dms-4D*) are 5 cM apart, which possibly contain two or more tightly linked genes or QTLs. Additional study is needed to explore whether such major effect coincident genomic region is due to tight linkage or pleotropic effect.

## Conclusions

There were discrepancies between our QTL results from the present and previous studies. First, we were not able to clearly confirm the flowering time and maturity QTLs on both 1B and 5B that were identified in our previous study using DArT and SSR markers. Second, the SNP and two functional markers provided us a better opportunity to uncover eight moderate effect and two major effect QTLs along with several other minor effect QTLs that were not identified in our previous study using SSR and DArT markers. The two major effect QTLs mapped on both chromosomes 2D and 4D. The QTL on 2D mapped adjacent to a well-known photoperiod response *Ppd-D1* gene and reduced flowering and maturity time up to 5 days but showed yield penalty by 436 kg ha^-1^. The QTL on 4D mapped adjacent to a well-known height reducing *Rht-D1* gene and reduced plant height on average by 13 cm, but increased maturity by 33 degree days. The coincident nature of the QTLs on 2D and 4D is very likely due to linkage, which may be determined by screening large numbers of recombinants to break up the linkage.

## Supporting Information

S1 Table Descriptive and F statistics. (XLSX)

S2 Table The position of the 10,342 single nucleotide polymorphic (SNP) markers integrated in the ‘Cutler’ x ‘AC Barrie’ linkage map. (XLSX)

S3 Table QTLs summary for both individual and combined environments. (XLSX)

S4 Table F statistics for the four coincident QTLs. (XLSX)

S5 Table Earliness *per se* QTLs on chromosomes 1B and 5B based on combined linkage map of DArT, SSR_and SNP markers from 131 RILs. (XLSX)
